# Myosite ossifiant circonscrite de la hanche: à propos d’un cas

**DOI:** 10.11604/pamj.2018.29.207.14126

**Published:** 2018-04-09

**Authors:** Abdoul Wahab Alassane Mohamed, Koini Moussa, Souna Badio Seyni, Zirbine Abassa Seyni, Akambi Sanoussi Kasoumou, Konguise Ziberou

**Affiliations:** 1 Service d'Orthopédie et de Traumatologie, Hôpital National du Niger, Niamey, Niger,; 2 Service d'Orthopédie et de Traumatologie, Hôpital Régional du Niger, Maradi, Niger,; 3 Service d'Orthopédie et de Traumatologie, Lamordé Hôpital National (CHU), Niamey, Niger,; 4 Service d'Orthopédie et de Traumatologie, Hôpital National du Niger, Zinder, Niger

**Keywords:** Myosite ossifiante, radiographie standard, hanche, Myositis ossificans, standard X-ray, hip

## Abstract

La myosite ossifiante circonscrite (MOC) est une affection bénigne, rare, caractérisée par une ossification hétérotopique localisée dans les tissus mous. Nous rapportons l’observation d’un adolescent de 15 ans se plaignant d’une douleur d’allure mixte à la hanche, aucun contexte traumatique n’a été évoqué à l’interrogatoire. Cette articulation avait un aspect inflammatoire et une tuméfaction non mobilisable adhérant au plan profond dont sa projection pariétale comblait le pli de l’aine. Les radiographies de la hanche ont visualisé un épaississement des parties molles de la hanche et des calcifications périarticulaires d’aspect flou. L’indication d’une biopsie exérèse des calcifications a été posée et réalisée. Le diagnostic confirmé par l’étude histologique d’un fragment biopsique. A six mois le patient était très satisfait du résultat fonctionnel de son membre. La reprise des activités sportives scolaires était autorisée avec succès.

## Abstract (en)

Myositis ossificans circumscripta (MOC) is a rare benign condition characterized by heterotopic ossification occurring in the soft tissues. We report the case of a 15-year old patient complaining of mixed hip pain. No trauma has been reported during the interview. The joint had inflammation appearance and a non-mobilizing swelling adhered to the deeper structure with parietal projection over the groin fold. Radiographs of the hip showed a thickening of the soft tissues of the hip and some periarticular calcifications with unclear appearance. Excisional biopsy of the calcifications was decided and performed. Diagnosis was confirmed by histological examination of the surgical specimen. At six months follow-up the patient was very satisfied with his functional results. Returning to school sports was authorized. The patient had successful outcome.

## Introduction

La myosite ossifiante circonscrite (MOC) est une affection bénigne souvent méconnue. Caractérisée par une ossification hétérotopique non tumorale des tissus mous. C’est une pathologie du sujet jeune, survenant le plus souvent après un traumatisme. Sa localisation est ubiquitaire, prédominante au niveau des ceintures et des membres. Son évolution spontanée est généralement favorable, conduisant à l’abstention thérapeutique [1]. Nous rapportons un cas de MOC survenant chez un adolescent de 15 ans.

## Patient et observation

**Figure 1:**
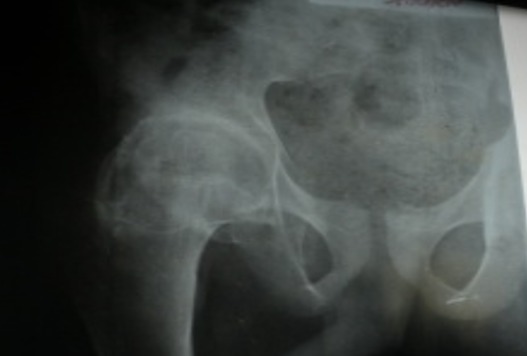
Image radiologique de calcifications péri articulaires à disposition annulaire de la hanche droite

**Figure 2:**
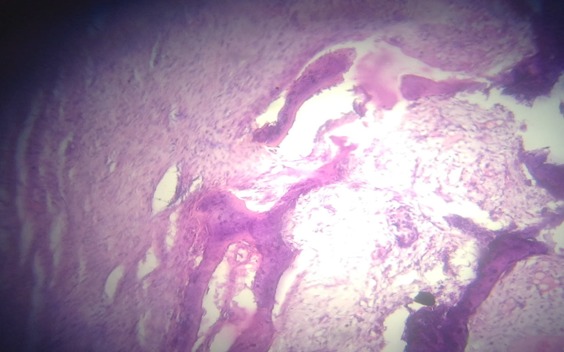
Coupe histologique de la pièce confirmant le diagnostic

**Figure 3:**
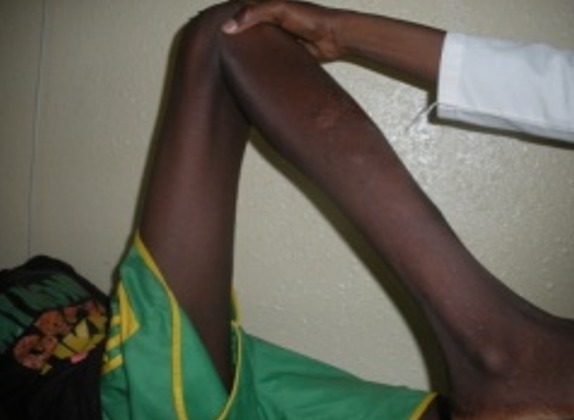
Restauration fonctionnelle du membre

Un jeune garçon âgé de 15 ans, sans antécédent pathologique particulier, consulte à l’HNL pour une douleur de la hanche droite qui évolue depuis plus d’un an, d’allure mixte (à la marche, lorsque le patient se relève d’une position assise et lors d’une station assise prolongée), sans notion de traumatisme. L’examen clinique avait retrouvé une boiterie d’esquive à la marche, tuméfaction dure, fixe, en regard de l’aine droite, une douleur vive à la palpation de la face antérieure de la hanche et à la mobilisation passive de la hanche, avec une flexion passive limitée à 40°. La radiographie a montré des calcifications péri-articulaires à disposition annulaire de la hanche droite (Figure 1). Le bilan biologique note un syndrome inflammatoire (une vitesse de sédimentation qui était très accélérée et la CRP était à deux chiffres). L’indication d’une biopsie-exérèse a été réalisée. Un abord antérieur selon Hueter a permis une bonne exposition, un jour excellent et une exérèse facile de la calcification. Les suites opératoires étaient simples. L’examen anatomopathologique de la pièce opératoire a confirmé le diagnostic de myosite ossifiante dans sa phase tardive (Figure 2). Il a aussi bénéficié d’un traitement médical à base d’antalgique anti-inflammatoire et de la kinésithérapie avec à sa sortie une bonne cicatrisation de la plaie. Au dernier recul à six mois, on a noté une bonne amélioration de la douleur et une flexion passive à 90° (Figure 3), puis la reprise des activités sportives scolaires a été autorisée à un an avec succès.

## Discussion

La MOC est une affection bénigne et rare. Une centaine d’observations sont rapportées dans la littérature [1]. Cette affection acquise est caractérisée par une ossification hétérotopique localisée due à la prolifération d’un tissu fibreux et a` la néoformation d’os et de cartilage dans les tissus mous [2, 3]. C’est une affection extrêmement rare qui touche surtout le sujet jeune sans prédominance de sexe [4]. Son diagnostic difficile à cause de multiples diagnostiques différentiels à éliminer surtout la myosite ossifiante progressive ou maladie de Munchmeyer et les autres maladies [2, 5]. Son traitement doit être conservateur (surveillance radio-clinique) car son involution est la règle et il n’y a pas de risque de malignité [2, 6, 7]. Elle peut se localiser dans n’importe quel muscle mais plus fréquemment au niveau des muscles volumineux des membres, dans notre cas il s’agit donc d’une localisation fréquente [7-10]. Deux éléments importants sont a` souligner et relatent du caractère bénin de la lésion: le respect de la corticale en regard de la masse et l’existence d’une ligne claire entre l’os et les parties molles. L’étude anatomopathologique de la tumeur met en évidence à partir de la quatrième semaine une architecture zonale en 3 couches concentriques très caractéristique de la MOC décrite par Ackerman [3, 9]: la zone centrale cellulaire, moins différenciée, renfermant des fibroblastes jeunes mais sans anarchie cellulaire ou mitose anormale. Une zone intermédiaire contenant des ostéoblastes et des dépôts ostéoides. Une zone périphérique externe, plus différenciée, formée de travées d’os mature. La pathogénie de la MOC reste encore mal élucidée [2, 6, 9-11]. En effet, de multiples facteurs comme le *Bone Morphogenic Protein-2* semblent jouer un role dans les phénomènes d’ossifications hétérotopiques [2, 12-15]. L’évolution est souvent favorable. La stabilisation spontanée des anomalies radiocliniques voire même la régression complète des lésions survient dans les 18 mois a` 3 ans après le début des signes.

## Conclusion

Maladie rarissime et bénigne, mais grave du fait de son gène fonctionnel, son diagnostic est surtout anatopathologique, l’imagerie joue un rôle très significatif et son traitement est symptomatique le plus souvent: rôle d’une abstention à la chirurgie.

## Conflits d’intérêts

Les auteurs ne déclarent aucun conflit d’intérêts.

## Contributions des auteurs

Tous les auteurs ont lu et approuvé la version finale du manuscrit.
